# Correlation of Hypertension and Proteinuria with Outcome in Elderly Bevacizumab-Treated Patients with Metastatic Colorectal Cancer

**DOI:** 10.1371/journal.pone.0116527

**Published:** 2015-01-20

**Authors:** Jaime Feliu, Antonieta Salud, Maria J. Safont, Carlos García-Girón, Jorge Aparicio, Ferran Losa, Carlos Bosch, Pilar Escudero, Enrique Casado, Monica Jorge, Uriel Bohn, Ramon Pérez-Carrión, Alberto Carmona, Ana B. Custodio, Joan Maurel

**Affiliations:** 1 Servicio de Oncología Médica, Hospital Universitario La Paz, Madrid, Spain; 2 Servicio de Oncología Médica, Hospital Arnau de Vilanova, Lleida, Spain; 3 Servicio de Oncología Médica, Hospital Universitario Clinico de Valencia, Valencia, Spain; 4 Servicio de Oncología Médica, Hospital General Yagüe, Burgos, Spain; 5 Servicio de Oncología Médica, Hospital La Fe de Valencia, Valencia, Spain; 6 Servicio de Oncología Médica, Hospital de La Creu Roja de L´Hospitalet, Barcelona, Spain; 7 Servicio de Oncología Médica, Hospital General de Valencia, Valencia, Spain; 8 Servicio de Oncología Médica, Hospital Clínico Universitario Lozano Blesa, Zaragoza, Spain; 9 Servicio de Oncología Médica, Hospital Infanta Sofía, Madrid, Spain; 10 Servicio de Oncología Médica, Complejo Hospitalario Xeral Cies, Vigo, Pontevedra, Spain; 11 Servicio de Oncología Médica, Hospital Universitario de Gran Canaria Doctor Negrin, Las Palmas de Gran Canaria, Spain; 12 Servicio de Oncología Médica, Hospital Universitario Quiron Madrid, Madrid, Spain; 13 Servicio de Hematología y Oncología Médica, Hospital General Universitario Morales Messeguer, Murcia, Spain; 14 Servicio de Oncología Médica, Hospital Clinic i Provincial de Barcelona, Barcelona, Spain; European Institute of Oncology, ITALY

## Abstract

**Background:**

Studies suggest a relationship between hypertension and outcome in bevacizumab-treated patients with metastatic colorectal cancer (mCRC). We performed a retrospective analysis of two phase II studies (BECA and BECOX) to determine if hypertension and proteinuria predict outcome in elderly patients with mCRC treated with bevacizumab.

**Patients and Methods:**

Patients ≥70 years of age received either capecitabine 1250 mg/m^2^ bid days 1–14 + bevacizumab 7.5 mg/kg day 1 every 21 days (BECA study) or capecitabine 1000 mg/m^2^ bid days 1–14 with bevacizumab 7.5 mg/kg and oxaliplatin 130 mg/m^2^ day 1 (BECOX study). The primary objective was to correlate hypertension and proteinuria with overall response rate (ORR), time to progression (TTP) and overall survival (OS). Secondary objectives included identification of risk factors associated with the development of hypertension and proteinuria and determining whether development of hypertension or proteinuria in the first 2 cycles was related to ORR, disease-control rate (DCR), TTP or OS.

**Results:**

In total, 127 patients (median age 75.5 years) were included in the study. Hypertension correlated with DCR and OS; proteinuria correlated with ORR and DCR. Proteinuria or hypertension in the first 2 cycles did not correlate with efficacy. Risk factors for hypertension were female gender (odds ratio [OR] 0.241; *P* = 0.011) and more bevacizumab cycles (OR 1.112; *P* = 0.002); risk factors for proteinuria were diabetes (OR 3.869; *P* = 0.006) and more bevacizumab cycles (OR 1.181; *P*<0.0001). Multivariate analysis identified as having prognostic value: baseline lactate dehydrogenase, haemoglobin, number of metastatic lesions and DCR.

**Conclusion:**

This analysis of two phase II studies suggests that hypertension is significantly correlated with OS but not with ORR and TTP, whereas proteinuria is correlated with ORR but not with OS and TTP. Both hypertension and proteinuria are associated with the duration of bevacizumab treatment and do not represent an independent prognostic factor.

## Introduction

Colorectal cancer (CRC) is the second most common cancer in Europe, with an estimated overall incidence of 447 per 100 000 population [1]. The incidence of CRC is strongly related to age, increasing sharply from the age of 50 years and reaching a peak at age >80 years [2]. In the UK, approximately 72% of CRC diagnoses between 2007 and 2009 were in people aged ≥65 years [2].

Treatment for elderly patients with metastatic colorectal cancer (mCRC) is complex and varies depending on the age, performance status, comorbidities and personal wishes of the patient. Recommended chemotherapy regimens for elderly patients comprise a fluoropyrimidine with or without oxaliplatin or irinotecan, as is the case for younger patients; however, real-life clinical studies have shown that monotherapy is more commonly given to older patients [3, 4]. Targeted agents, such as bevacizumab, are also recommended [5] but are less commonly used in elderly vs younger patients [3].

Several studies have suggested a relationship between the development of hypertension, a common side effect of bevacizumab treatment, and outcome in bevacizumab-treated patients with mCRC [6–8], melanoma [9] or lung cancer [10]. Proteinuria is also widely reported in bevacizumab-treated patients and potential links with outcome are also of interest.

As the incidences of hypertension and proteinuria increase with age, we undertook a retrospective analysis of two studies performed in elderly patients with mCRC—BECA ([11]; EudraCT 2005–002808–42) and BECOX ([12]; NCT01067053)—to determine if these side effects are indicative of outcome in elderly bevacizumab-treated patients.

## Materials and Methods

### Study design

This was a retrospective pooled analysis of two prospective phase II clinical studies performed in elderly patients with mCRC in Spain: BECA ([11]; EudraCT 2005–002808–42) and BECOX ([12]; NCT01067053). In brief, patients were ≥70 years of age, had histologically confirmed but unresectable mCRC, Eastern Cooperative Oncology Group (ECOG) performance status 0–2 and measurable disease.

Exclusion criteria included central nervous system metastases, clinically significant cardiac disease, clinical use of full-dose anticoagulants or thrombolytics, and major surgical procedures within 28 days before study entry.

Both original studies were approved by the appropriate Institutional Review Boards (IRB) and were conducted according to the principles expressed in the Declaration of Helsinki. Informed consent, written or oral, was obtained from the participants.

### Treatment

In the BECA study, treatment consisted of bevacizumab 7.5 mg/kg on day 1 every 3 weeks plus capecitabine 1250 mg/m^2^ (950 mg/m^2^ for patients with creatinine clearance of 30–50 mg/min) twice daily (bid) for 2 weeks followed by 1 week of rest, for a minimum of 3 cycles. In the BECOX study, treatment consisted of bevacizumab 7.5 mg/kg plus oxaliplatin 130 mg/m^2^ on day 1 every 3 weeks plus capecitabine 1000 mg/m^2^ bid for 2 weeks followed by 1 week of rest; oxaliplatin was discontinued after 6 cycles.

### Measurement of blood pressure and proteinuria

Blood pressure measurements were performed by a nurse on patients in a resting position (after 10 minutes’ rest) at baseline and before each bevacizumab infusion. If hypertension was observed, a repeat measurement at an unscheduled visit was requested for confirmation. In such cases, the maximum value was used for this analysis. Hypertension was defined as grade 1: asymptomatic, transient increase in blood pressure of >20 mmHg or to >150/100 mmHg if previously within normal limits, no treatment required; grade 2: recurrent or persistent increase of >20 mmHg or to >150/100 mmHg if previously within normal limits, monotherapy may be required; grade 3: hypertension requiring more than one drug or more intensive therapy than previously; and grade 4: live-threatening consequences (eg, hypertensive crisis).

Proteinuria was measured at baseline and before each bevacizumab infusion. Proteinuria was defined as + (0.15–1.0 g 24h^-1^), ++/+++ (>1.0–3.5 g 24 h^-1^), and ++++ (>3.5 g 24 h^-1^).

### Statistical and multivariate analyses

The primary objective of this analysis was to correlate hypertension and proteinuria with overall response rate (ORR), time to progression (TTP) and overall survival (OS). Secondary objectives included determining the prevalence of hypertension and proteinuria; investigating the relationship between hypertension and proteinuria and the number of cycles; identification of risk factors associated with the development of hypertension and proteinuria and determining whether development of hypertension or proteinuria in the first 2 cycles was related to ORR, disease-control rate (DCR), TTP or OS.

Quantitative variables were characterised using centralisation and dispersion (mean, median, standard deviation [SD], minimum and maximum). Qualitative variables were described by absolute and relative frequencies. Survival analyses were performed using Kaplan–Meier methodology with 95% confidence intervals (CIs). Qualitative variables were compared using chi-squared or Fisher tests as appropriate.

A logistic regression analysis was performed using development of hypertension as the dependent variable and the following factors as independent variables: previous hypertension (no vs yes); diabetes (no vs yes); thromboembolic disease (no vs yes); baseline BMI (normal vs overweight/obese); cumulative dose of bevacizumab (low cumulative dose [≤1229 mg] vs medium–low cumulative dose [>1229 mg and ≤3060 mg] vs medium–high cumulative dose [>3060 and ≤5400 mg] vs high cumulative dose [>5400 mg]); age; gender (female vs male); creatinine clearance (baseline creatinine clearance in ml.min^-1^); and number of cycles of bevacizumab. Bivariate logistic regressions were performed with hypertension and proteinuria as dependent variables and the factors listed above as independent variables. Factors showing statistical significance (P<0.20) were built into a multivariate model.

To identify factors associated with OS, a Cox regression analysis was performed using OS as the dependent variable and the following as independent variables: development of grade 1–4 hypertension (no vs yes); gender (female vs male); ECOG performance status (0 vs 1 vs 2); age; baseline lactate dehydrogenase (LDH); baseline carcinoembryonic antigen (CEA); baseline platelets; baseline haemoglobin; baseline leucocytes; type of treatment (bevacizumab + capecitabine + oxaliplatin vs bevacizumab + capecitabine) and number of metastatic lesions. A bivariate Cox regression was performed with OS as the dependent variable and the above factors as independent variables. Factors showing statistical significance (P<0.20) were built into the multivariate model.

Data were analysed using SPSS version 17.0 (SPSS Inc, Chicago IL, USA).

## Results

### Patients

The analysis population comprised 127 patients, 59 from the BECA study and 68 from the BECOX study recruited between 1 August 2006 and 1 March 2012. Patient characteristics at baseline are summarised in Table 1; 68 patients had pre-existing hypertension and, of these, hypertension worsened in 11 patients; in the 59 patients (15%) that did not have pre-existing hypertension, 9 patients developed hypertension during the study.

**Table 1:** Patient characteristics at baseline.

**Characteristic**	**Total population**	**BECA**	**BECOX**
	**(n = 127)**	**(n = 59)**	**(n = 68)**
**Gender, n (%)**			
Male	78 (61)	34 (58)	44 (65)
Female	49 (39)	25 (42)	24 (35)
**Age, years**			
Median	76	75	76
Range	70–88	73–79	71–85
**ECOG performance status, n (%)**			
0	59 (45)	26 (44)	32 (47)
1	66 (52)	31 (53)	36 (53)
2	2 (2)	2 (3)	0
**Comorbid conditions (previous or current), n (%)**			
Hypertension	68 (54)	36 (61)	32 (47)
Diabetes	35 (28)	19 (32)	16 (24)
Thromboembolic disease	7 (6)	3 (5)	4 (6)

Abbreviation: ECOG = Eastern Cooperative Oncology Group.

Patients received a median of 6 bevacizumab cycles (range 1–34) and a median cumulative bevacizumab dose of 3060 mg (range 320–22 005). Median TTP was 10.9 months (95% CI 8.9–12.8); median OS was 20.1 months (95% CI 16.0–24.2) and ORR was 39.4% (95% CI 31.0–48.5).

### Hypertension

Twenty patients (16%) developed hypertension during the study (11 with pre-existing hypertension and 9 without pre-existing hypertension); this was moderate to severe in 12 patients (9%). Four patients (3%) had hypertension during the first 2 cycles. Among the 52 patients who had treatment lasting ≥6 months, 16 (31%) developed hypertension. Patients who developed hypertension had a median of 12 (range 2–33 cycles) bevacizumab cycles compared with 5 cycles (range 1–34) in those who did not (*P*<0.0001).

Risk factors associated with the development of hypertension in the bivariate analysis were cumulative bevacizumab dose (*P* = 0.012), gender (*P* = 0.003) and number of bevacizumab cycles (*P* = 0.001). Only gender and number of bevacizumab cycles were statistically significant in the multivariate analysis: men had a lower risk of developing hypertension than women (odds ratio [OR] 0.241; 95% CI 0.081–0.717; *P* = 0.011), as did patients who had fewer bevacizumab cycles (OR 1.112; 95% CI 1.039–1.191; *P* = 0.002). Further analysis to assess whether gender was a potential effect modifier or confounding factor in the relationship between development of hypertension and number of bevacizumab cycles received determined that this was not the case.

### Hypertension and outcome

**Figure 1:**
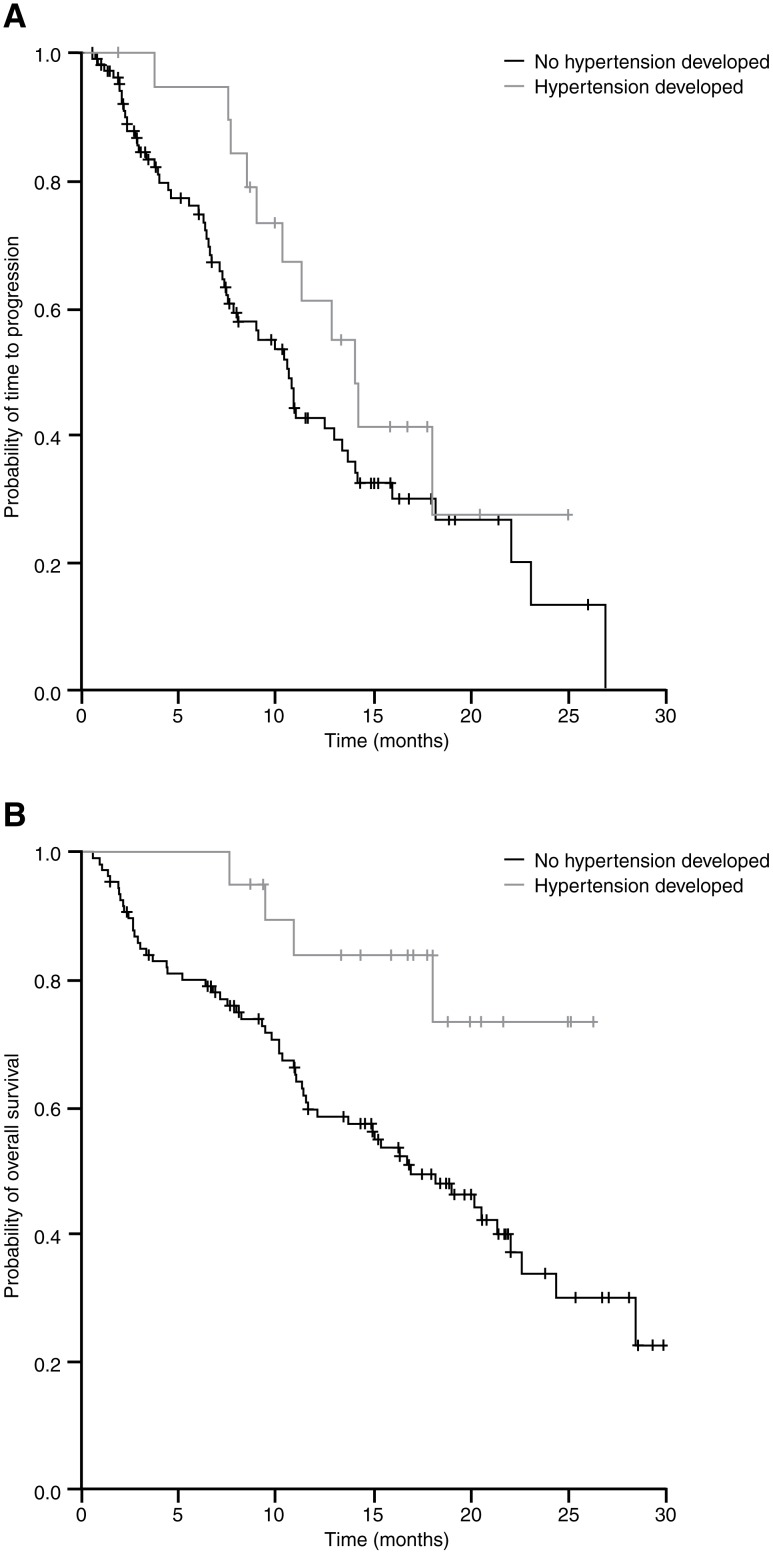
Kaplan–Meier curves of (A) time to progression and (B) overall survival in patients who did and did not develop hypertension during treatment. Patients with hypertension during the study had significantly greater median OS than those with no hypertension. Median TTP was numerically greater in patients with hypertension but this difference did not reach statistical significance.

Patients with hypertension during the study had significantly higher DCR and median OS than those with no hypertension (Table 2, Fig. 1). ORR and median TTP were numerically higher in patients with hypertension, but these differences were not statistically significant.

**Table 2:** Correlation between hypertension and proteinuria and response to treatment in the BECA and BECOX studies.

	**Hypertension**			**Proteinuria**		
**Outcome**	**Yes (n = 20)**	**No (n = 107)**	***P* value**	**Yes (n = 77)**	**No (n = 50)**	***P* value**
Median OS, months	NR	16.9	0.012^a^	22.0	20.1	0.211^a^
Median TTP, months	14.0	10.6	0.174^a^	13.0	7.4	0.063^a^
ORR, %	50	37	0.325^b^	47	28	0.042^b^
DCR, %	95	71	0.024^b^	86	58	0.001^b^

Abbreviation: DCR = disease-control rate; NR = not reached; ORR = overall response rate; OS = overall survival; TTP = time to progression.

^a^Log-rank test.

^b^Fisher’s exact test

When more severe hypertension (grade 2–4; n = 12) was considered, ORR (58% for patients with moderate to severe hypertension vs 37% for those with grade 1 or no hypertension), DCR (92% vs 73%), TTP (14.2 vs 10.8 months) and OS (not reached vs 18.9 months) were numerically higher in patients with hypertension although these differences were not statistically significant.

There was no correlation between the development of hypertension in the first 2 treatment cycles (n = 4) and treatment outcome (TTP of 11.3 months for patients developing hypertension vs 10.8 months for those not developing hypertension [*P* = 0.659] and OS (not reached vs 20.1 months [*P* = 0.468]).

In patients with no prior hypertension (n = 59), those who developed hypertension during the study (n = 9) had a numerically higher ORR (67% vs 36% for those who did not), DCR (100% vs 78%) and longer TTP (18.0 vs 10.6 months); none of these differences was statistically significant. Those who did not develop hypertension during the study had longer OS than those who did (20.5 vs 18.0 months); this difference was not statistically significant.

### Proteinuria

A total of 77 patients (61%) had proteinuria during the study; this was moderate to severe in 16 patients (13%). Proteinuria occurred during the first 2 cycles in 45 patients (35%). Among the 52 patients who had ≥6 months of bevacizumab treatment, 41 (79%) developed proteinuria. The median number of bevacizumab cycles administered to patients who developed proteinuria was 8 (range 1–34) compared with 4 cycles (range 1–25) in those with no proteinuria (*P*<0.0001).

Risk factors associated with the development of proteinuria in the bivariate analysis were diabetes (*P* = 0.022), cumulative bevacizumab dose (*P* = 0.001), age (*P* = 0.159) and number of bevacizumab cycles (*P*<0.0001). In the multivariate analysis, only patients with diabetes (OR 3.869; 95% CI 1.470–10.184; *P* = 0.006) and those receiving more bevacizumab cycles (OR 1.181; 95% CI 1.083–1.288; *P*<0.0001) had an increased risk of developing proteinuria.

### Proteinuria and outcome

**Figure 2:**
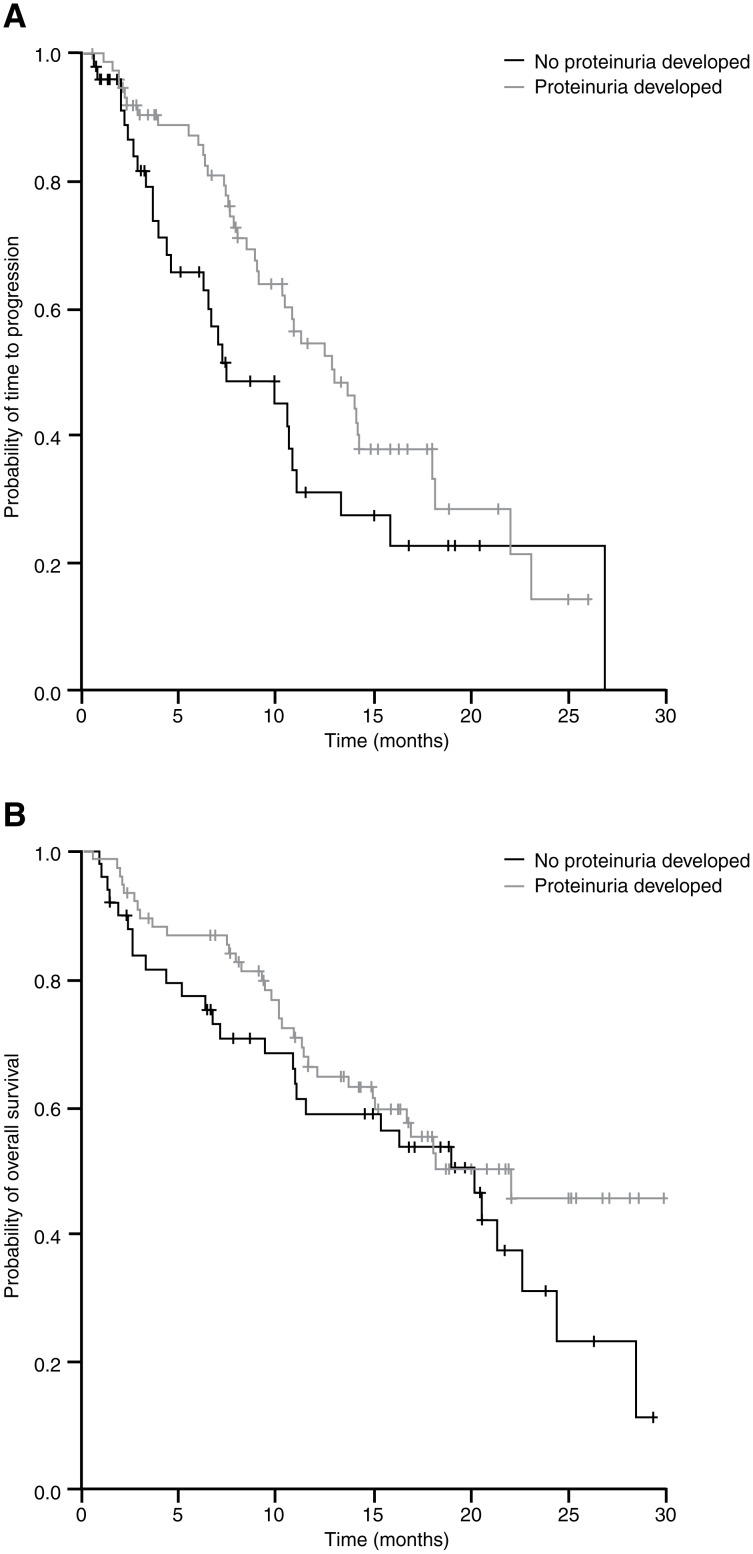
Kaplan–Meier curves of (A) time to progression and (B) overall survival in patients who did and did not develop proteinuria during treatment. Patients who had proteinuria during the study had numerically, but not statistically significantly, greater TTP and OS compared with those patients who did not have proteinuria.

Patients who had proteinuria during the study had a higher ORR and DCR than those who did not (*P* = 0.042 and *P* = 0.001, respectively). TTP and OS were numerically but not statistically significantly higher in patients who had proteinuria (Table 2; Fig. 2).

When moderate-to-severe proteinuria was considered (++, +++, ++++; n = 16), ORR (56% for patients with moderate-to-severe proteinuria vs 37% for those with mild or no proteinuria), DCR (94% vs 72%) and OS (22.0 vs 20.1 months) were numerically but not statistically significantly higher in patients with proteinuria. A trend towards correlation of TTP and moderate-to-severe proteinuria was observed (22.0 vs 10.4 months; *P* = 0.051). There was no correlation between development of moderate-to-severe proteinuria during the first 2 treatment cycles (n = 6) and any of the outcomes studied (TTP of 10.9 months for both groups [*P* = 0.559] and OS of 20.5 months for patients not developing proteinuria vs 9.2 months for those developing proteinuria [*P* = 0.259]).

No correlation was observed between proteinuria and hypertension or between oxaliplatin use and either hypertension or proteinuria.

### Multivariate analysis of survival

The following factors were statistically significantly associated with OS in the bivariate analysis: development of hypertension (*P* = 0.018); baseline LDH (*P*<0.0001); haemoglobin (*P* = 0.011); leucocytes (*P*<0.0001); number of metastatic lesions (*P* = 0.002); and DCR (*P*<0.0001).

In the multivariate analysis, the following groups were identified as having a lower risk of death: patients with lower baseline LDH (HR 1.0004; 95% CI 1.000–1.001; *P* = 0.002); patients with higher baseline haemoglobin (HR 0.773; 95% CI, 0.635–0.941; *P* = 0.01); fewer metastatic lesions at baseline (HR 1.165; 95% CI 1.0441–1.300; *P* = 0.006); and DCR (HR 0.224; 95%CI 0.120–0.419; *P*<0.0001).

## Discussion

Unlike other targeted agents for which biomarkers indicating lack of response to treatment have been identified, it is not yet possible to predict which patients will respond to bevacizumab. As a result, other markers of response have been investigated, including hypertension and proteinuria, both of which are common side effects of treatment with bevacizumab.

In the present analysis, patients with any-grade hypertension occurring any time during the study had significantly higher OS and DCR than those with no hypertension, in line with previous studies [6–8]. Early and more severe hypertension were not significantly associated with outcome. When patients with pre-existing hypertension were excluded from the analysis, the relationship between hypertension and treatment was no longer statistically significant. Our findings contradict those of other studies suggesting that early hypertension [10, 13] and more severe hypertension are important predictive factors [6] and agree with others that observed no correlation between early hypertension and outcome [14, 15]. In our study, the development of hypertension was associated with female gender and the number of administered cycles of bevacizumab. Although in the univariate analysis the hypertension was associated with survival, the multivariate analysis failed to confirm this relationship. However, DCR, LDH, haemoglobin and the number of metastatic sites did demonstrate prognostic value. This suggests that in this analysis the development of hypertension during treatment with bevacizumab has no independent prognostic value, but probably depends on the DCR which, in turn, determines the likely duration of treatment with bevacizumab, the cumulative dose, and the risk of experiencing hypertension.

The development of proteinuria was associated with significantly better ORR and DCR in the present analysis. There was no significant association between any-grade proteinuria with OS or TTP, although a trend towards a correlation between more severe proteinuria and TTP was observed. The potential relationship between outcome and proteinuria has been less widely studied than the link between outcome and hypertension. That said, case reports have described prolonged survival in bevacizumab-treated patients with proteinuria with or without hypertension [16, 17], and a recent study by Berruti et al reported proteinuria (all grades) to correlate with longer progression-free survival (p = 0.017) in patients with metastatic well-to-moderately differentiated neuroendocrine tumors receiving bevacizumab plus octreotide and metronomic capecitabine [18]. These reports and our findings indicate that proteinuria might be a marker for the activity of bevacizumab. In contrast, however, Iwasa et al saw no correlation between proteinuria and survival in patients with mCRC [19]. Further studies in larger patient cohorts are needed to investigate the role of proteinuria as a biomarker for bevacizumab activity.

The incidence of proteinuria in the present study was higher than reported elsewhere [20–22]. This may be due to factors such as incidence of diabetes and cumulative bevacizumab dose administered in those studies; for example, the incidence of diabetes in the present study was higher than that reported in the boxe study [22]. In contrast, the incidence of hypertension was lower than observed in previous studies of bevacizumab in elderly patients [20–22].

Anti-VEGF induced hypertension appears to be related to suppression of nitric oxide production in the endothelial cells [23] and proteinuria results from inhibition of paracrine VEGF signaling between VEGF-producing glomerular podocytes and adjacent endothelial cells [24]. Inhibition of podocyte-endothelial cell VEGF signaling, whether through genetic or pharmacological means, causes endotheliosis, thrombotic microangiopathy, and the narrowing of capillary lumen seen in patients with albuminuria receiving VEGF-targeted therapies [24]. The relationship between bevacizumab-related hypertension and proteinuria with treatment response may also have a genetic explanation. Studies have identified genetic variants of VEGFA and VEGFR1 potentially associated with response to bevacizumab [25, 26]. These genetic variants, which increase the sensitivity of endothelial cells to the effects of anti-VEGF agents, may be linked to an increased risk of toxicity. Therefore, patients developing hypertension or proteinuria during bevacizumab treatment may be carriers of such variants. Indeed, VEGF polymorphisms associated with hypertension have been identified in patients treated with sunitinib [27] and bevacizumab [28]. We believe this hypothesis warrants further investigation in prospective clinical trials.

Some potential limitations of this retrospective study should be considered. Despite the fact that two studies were pooled, some of the analyses (development of hypertension or proteinuria during the first 2 treatment cycles) were performed on small numbers of patients. Blood pressure was taken before each cycle as recommended in the Avastin Summary of Product Characteristics [29]; however, others have measured blood pressure twice daily [13], and blood pressure may also be taken continuously using ambulatory monitoring. In addition, there may have been some differences between the two studies in how blood pressure was monitored.

In conclusion, this analysis of pooled data from the BECA and BECOX studies suggests that hypertension is significantly correlated with OS but not with ORR and TTP, whereas proteinuria is correlated with ORR but not with OS and TTP. The presence of hypertension or proteinuria in elderly bevacizumab-treated patients with mCRC are related with the duration of bevacizumab treatment and do not represent an independent prognostic factor.
